# Cellular expression of epigenetic markers and oxidative stress in periodontitis lesions of smokers and non‐smokers

**DOI:** 10.1111/jre.13030

**Published:** 2022-06-29

**Authors:** Carlotta Dionigi, Lena Larsson, Julia C. Difloe‐Geisert, Nicola U. Zitzmann, Tord Berglundh

**Affiliations:** ^1^ Department of Periodontology, Institute of Odontology, The Sahlgrenska Academy University of Gothenburg Gothenburg Sweden; ^2^ Department of Periodontology, Endodontology and Cariology, University Center for Dental Medicine University of Basel Basel Switzerland; ^3^ Department of Reconstructive Dentistry, University Center for Dental Medicine University of Basel Basel Switzerland

**Keywords:** biopsy, epigenetics, histology, immunohistochemistry, inflammation, periodontitis, smoking

## Abstract

**Objective:**

To evaluate differences in the cellular expression of epigenetic markers and oxidative stress in periodontitis lesions between current smokers and non‐smokers.

**Background:**

Tobacco smoking is recognized as one of the major risk factors for periodontitis. However, the mechanisms by which smoking affects the progression of the disease remain to be determined.

**Methods:**

Twenty‐five current smokers and 21 non‐smokers with generalized severe periodontitis were included. From each patient, one soft tissue biopsy from a periodontitis site was harvested and prepared for histological analysis. The infiltrated connective tissue (ICT) was selected as the region of interest to assess the cellular expression of epigenetic markers and reactive oxygen/nitrogen species (RONS) by immunohistochemistry.

**Results:**

Although the ICT of smokers and non‐smokers did not differ in size or in the expression of markers for DNA damage or oxidative stress, current smokers presented with significantly lower area proportions and densities of cells positive for the epigenetic markers DNMT1 and AcH3. In addition, periodontitis lesions in current smokers presented with a diminished antimicrobial activity, as indicated by significantly lower densities and area proportions of NOX2‐ and iNOS‐positive cells.

**Conclusions:**

Components of the host response and epigenetic mechanisms in periodontitis lesions in smokers are downregulated as opposed to lesions of non‐smokers.

## 1 INTRODUCTION

Periodontitis is a chronic multifactorial inflammatory disease characterized by progressive destruction of the supporting tissues around teeth.^1^ It is estimated that severe periodontitis occurs in 7.4% of the adult population, being the sixth most common human disease.^2^, ^3^ While associations between complex dysbiotic biofilms and the onset, progression, and sustention of the non‐resolving inflammatory process of periodontitis have been demonstrated,^4^ the role of environmental and genetic/epigenetic factors is not fully understood.^5^ Although results from multiple cross‐sectional and longitudinal studies have identified tobacco smoking as one of the major risk factors for periodontitis,^6^, ^7^, ^8^, ^9^ the mechanisms by which smoking affects the progression of periodontitis remain to be determined.

Chromatin arrangement is one of the main features of epigenetics and regulates gene expression without altering the DNA sequence.^10^ DNA methyltransferases (DNMTs) are a family of enzymes that add a methyl group to cytosine‐phosphate‐guanine (CpG) islands on the DNA structure. This process is known as DNA methylation and induces the inactivation of DNA transcription, leading to gene silencing. Other epigenetic mechanisms involve the regulation of histones, that is, the structural proteins around which the DNA helix coils within the nucleosome. Acetyl groups on the histone tail may be removed or added by histone acetyltransferases (HATs) and histone deacetylases (HDACs).

Studies have reported on differences in DNA‐methylation levels in whole blood samples among smokers, former smokers, and non‐smokers.^11^ In addition, DNA methylation may serve as a long‐term marker for exposure to tobacco smoke and smoking‐associated chronic disease.^12^, ^13^, ^14^ Few studies, however, have analyzed the association between epigenetic mechanisms and periodontitis in smokers and non‐smokers. Results from a pilot study indicated differences in DNA methylation in gingival biopsies between smokers and non‐smokers.^15^

Studies have shown that chemical substances in tobacco products generate genotoxic effects on the host immune response.^16^, ^17^ Thus, diminished chemotaxis and phagocytosis have been identified in neutrophils together with enhanced levels of markers for oxidative stress and production of reactive oxygen species (ROS).^18^, ^19^ NADPH‐oxidases (NOX) and nitric oxide synthases (NOS) are two families of enzymes produced by neutrophils and macrophages, and contribute to the formation of superoxide^20^ (O_2_^−^) and nitric oxide^21^ (NO). The antimicrobial function of these reactive oxygen and nitrogen species (RONS) is to eliminate microorganisms during phagocytosis. Thus, an excessive and prolonged production of RONS in chronic inflammation can generate cytotoxic effects, oxidative damage to DNA and lead to tissue breakdown.^22^

In a study on human biopsy material representing sites with severe periodontitis in current smokers and non‐smokers, densities of vascular units and inflammatory cells were assessed.^23^ While no differences in phenotype markers of inflammatory cells were detected between the two groups of samples, periodontitis lesions in smokers presented with fewer but larger vessels than lesions in non‐smokers. The aim of the present study was to further analyze the histological material presented by Schmidt et al.^23^ with a specific focus on differences in DNA methylation status and histone modification levels, and cellular expression of oxidative stress and reactive oxygen/nitrogen species between current smokers and non‐smokers.

## 2 MATERIAL AND METHODS

Forty‐six patients affected by generalized severe periodontitis [defined by radiographic bone loss ≥30% and probing pocket depth (PPD) ≥ 5 mm with bleeding on probing (BoP) in ≥25% of teeth] were recruited from the Department of Periodontology, Endodontology and Cariology, and from the undergraduate clinic at the School of Dental Medicine (University Centre for Dental Medicine, University of Basel,). The study protocol was approved by the local human institutional review board of the University of Basel, Switzerland (EK: 159/06).

Details on the recruitment of patients, inclusion/exclusion criteria and biopsy sampling procedures were previously reported by Schmidt et al.^23^ In brief, subjects were divided into two different groups depending on their smoking status. One group comprised 25 current smokers (52% women, mean age 48.5 ± 9.2 years, range 33–69 years) who had smoked ≥10 cigarettes per day for at least 3 years and/or to a level corresponding to ≥1.5 pack‐years according to self‐reported smoking status. The second group consisted of 21 current non‐smokers (57% women, mean age 53.7 ± 12.7 years, range 35–76 years) who never smoked or had smoked <100 cigarettes in their lifetime. Among the 21 current non‐smoking patients, eight subjects were former smokers who quit smoking at least 5 years prior to enrollment (mean time 23.7 ± 12.8 years, range 5–45 years).

Before enrolment, all subjects received information about the study and signed an informed consent form. Clinical and radiographic examinations were performed. Salivary samples were used to measure cotinine levels to confirm the self‐reported tobacco use before initiation of the study (Appendix S1).

The patients were classified as generalized stage III or stage IV periodontitis according to the 2017 World Workshop on Classification on Periodontal and Peri‐implant Diseases and Conditions.^1^ The patients of the smoking group were, in addition, graded to the C category, while non‐smokers were graded either to the B or the C category. From each patient, one biopsy from a diseased site (probing pocket depth ≥6 mm, clinical attachment loss ≥6 mm, and bleeding on probing: Appendix S2) was collected under local anesthesia before initiation of the non‐surgical periodontal therapy. After dissection, the tissue samples were rinsed in saline and placed in a 4% formaldehyde solution for at least 24 h.

### 2.1 Immunohistochemistry

After dehydration and embedding in paraffin, the biopsies were stored at room temperature. From each tissue portion, 5 μm‐thick sections were produced using a microtome, dewaxed and incubated in DIVA antigen‐retrieval solution (Biocare Medical,) at 60°C overnight. Following blocking of endogenous peroxidase and application of 4% bovine serum albumin (BSA), the sections were incubated with a primary antibody followed by incubation with Envision horseradish peroxidase (HRP)‐labeled polymer (Agilent,) for 30 min. Positive cells were detected using 3,3’ Diaminobenzidine (DAB) substrate (Agilent). The antibodies used for the immunohistochemical preparations and their dilutions are presented in Table 1. In brief, DNA methylation levels were identified by the DNMT1 and TET2 markers, while the histone acetylation levels were measured using the AcH3, AcH4, HDAC1, and HDAC2 markers. The γ‐H2AX marker was used to identify DNA‐damaged sites caused by double‐stranded breaks, while the 8‐OHdG marker was used to estimate the DNA damage caused by oxidative stress. The cellular expression of RONS (iNOS, NOX2) was also measured. Counterstaining was performed with hematoxylin. Finally, the sections were mounted and cover‐slipped. Negative controls were produced without the addition of the primary antibodies for each staining.

**TABLE 1:** Description of the antibodies used in the immunohistochemical analysis

Antibody	Type	Dilution (Time)	Target	Isotype	Source
DNMT1	Mouse monoclonal	1:5 (ON)	DNA methylation	IgG1k	Santa Cruz Biotechnology, Dallas, TX, USA
TET2	Mouse monoclonal	1:50 (1 h)	DNA demethylation	IgG1k	Active Motif, Waterloo, Belgium
AcH3	Rabbit monoclonal	1:500 (1 h)	Histone 3 acetylation	IgG	Cell Signalling Technology, Danvers, MA, USA
AcH4	Rabbit monoclonal	1:800 (1 h)	Histone 4 acetylation	IgG	Abcam, Cambridge, UK
HDAC1	Rabbit polyclonal	1:500 (1 h)	Histone deacetylation	IgG	Abcam, Cambridge, UK
HDAC2	Rabbit monoclonal	1:100 (1 h)	Histone deacetylation	IgG	Abcam, Cambridge, UK
γ‐H2AX	Rabbit polyclonal	1:100 (1 h)	DNA double‐strand breaks	IgG	Active Motif, Waterloo, Belgium
8‐OHdG	Mouse monoclonal	1:8000 (ON)	ROS oxidative stress	IgG2b	GeneTex, Irvine, CA, USA
iNOS	Rabbit polyclonal	1:10 (ON)	Antimicrobial NO	IgG	ThermoFisher Scientific (Invitrogen), Waltham, MA, USA
NOX2	Mouse monoclonal	1:500 (ON)	Antimicrobial NADPH oxidase	IgG1	Abcam, Cambridge, UK

Abbreviations: Overnight (ON); DNA‐methyltransferase 1 (DNMT1); Ten‐eleven‐translocation 2 (TET2); Acetyl‐histone H3 (AcH3); Acetyl‐histone H4 (AcH4); Histone deacetylase 1 (HDAC1); Histone deacetylase 2 (HDAC2); Phosphorylated H2A family member X (γ‐H2AX); 8‐hydroxyguanosine (8‐OHdG); Nitric oxide (NO); Inducible nitric oxide synthase (iNOS); Nicotinamide adenine dinucleotide phosphate (NADPH); NADPH‐oxidase 2 (NOX2); Reactive oxygen species (ROS).

### 2.2 Histological analysis

Qualitative and quantitative histological examinations of the inflammatory cell infiltrates were performed under a light microscope (Leitz DM‐RBE microscope, Leica,). Each section was captured with the Glissando Desktop Scanner (Objective Imaging Inc.,) and transferred to a computer equipped with the computerized image analysis software Image‐Pro Premier (IPP, ver. 10; Media Cybernetics Inc.,). The analysis was performed by one trained investigator (C.D) who was blinded to the origin of the samples. The infiltrated connective tissue (ICT) was depicted and outlined with a mouse cursor as the region of interest (ROI). The smart segmentation tool of the IPP software was used to identify each cell marker, using a differential method analysis of color, intensity, morphology, and size. Thus, the total area occupied by positive cells was assessed for each marker and its percentage area relative to the total area of the ICT was calculated. In addition, the average cell size for each cell marker category was assessed. The number of positive cells in the ICT was computed using the data from the ROI total area, the average cell size, and the total area occupied by the positive cells. Cell numbers were expressed as total number and density of cells (number of cells/mm^2^) within the ROI.

### 2.3 Data analysis

The power calculation was based on assessments of cell phenotypes as reported previously.^23^ Thus, a minimum of 20 patients per group (current smokers and non‐smokers) would be required to detect a difference of 4% in area proportions of positive cells, based on a standard deviation of 4.5, a power of 80% and *α* = 0.05. Statistical analyses were performed using the SPSS software (SPSS Statistics 24.0.0.0; SPSS Inc.,).

Mean values and standard deviations were calculated for each variable, using the patient as the experimental unit. Differences between the two groups (current smokers and non‐smokers) were analyzed using the Mann–Whitney *U*‐test for independent variables. The null hypothesis was rejected at *p* < .05.

## 3 RESULTS

**FIGURE 1:**
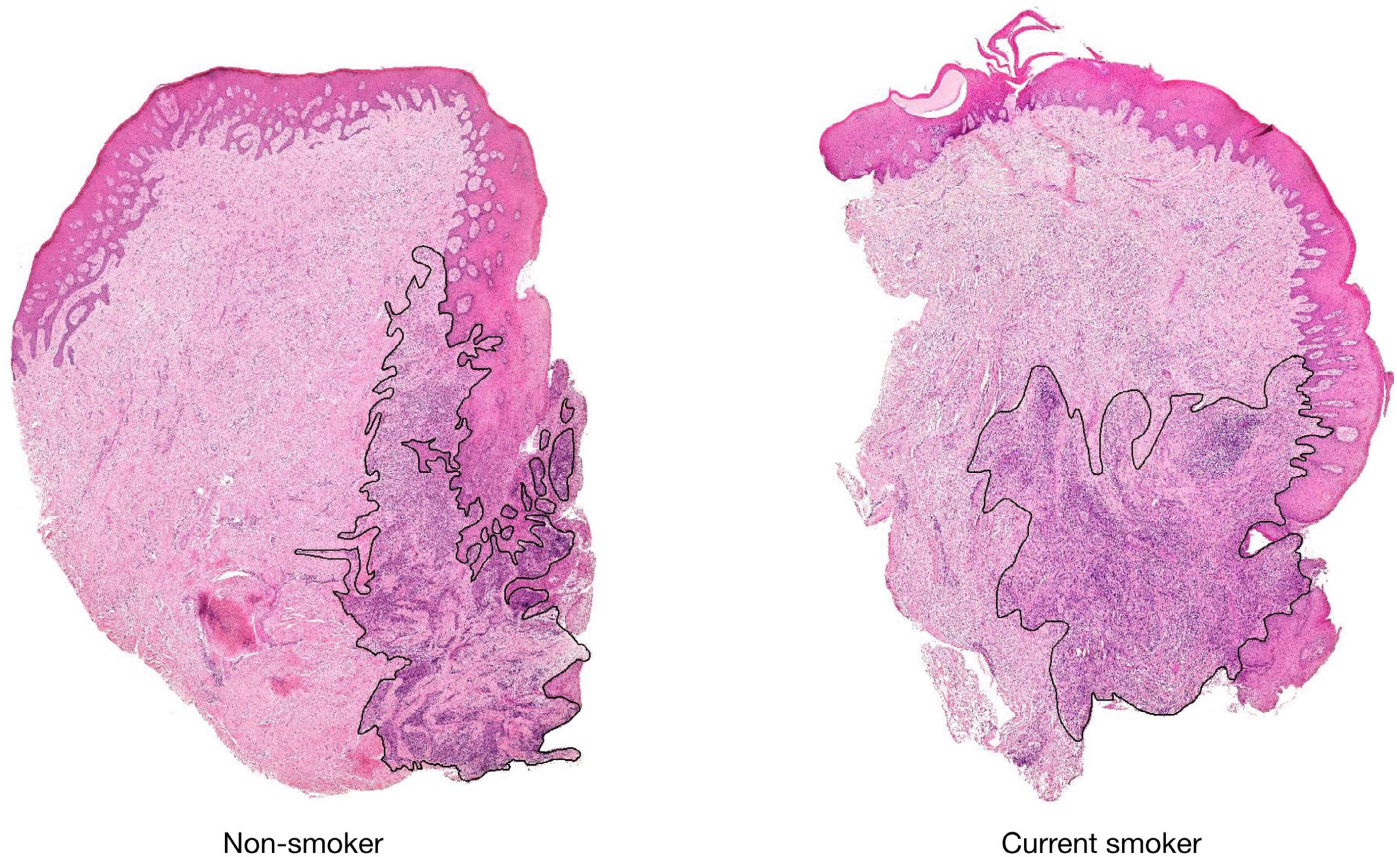
Histological sections stained with hematoxylin and eosin from periodontitis sites in a non‐smoker and a current smoker. Infiltrated connective tissue (ICT) outlined in black. Magnification ×20

**FIGURE 2:**
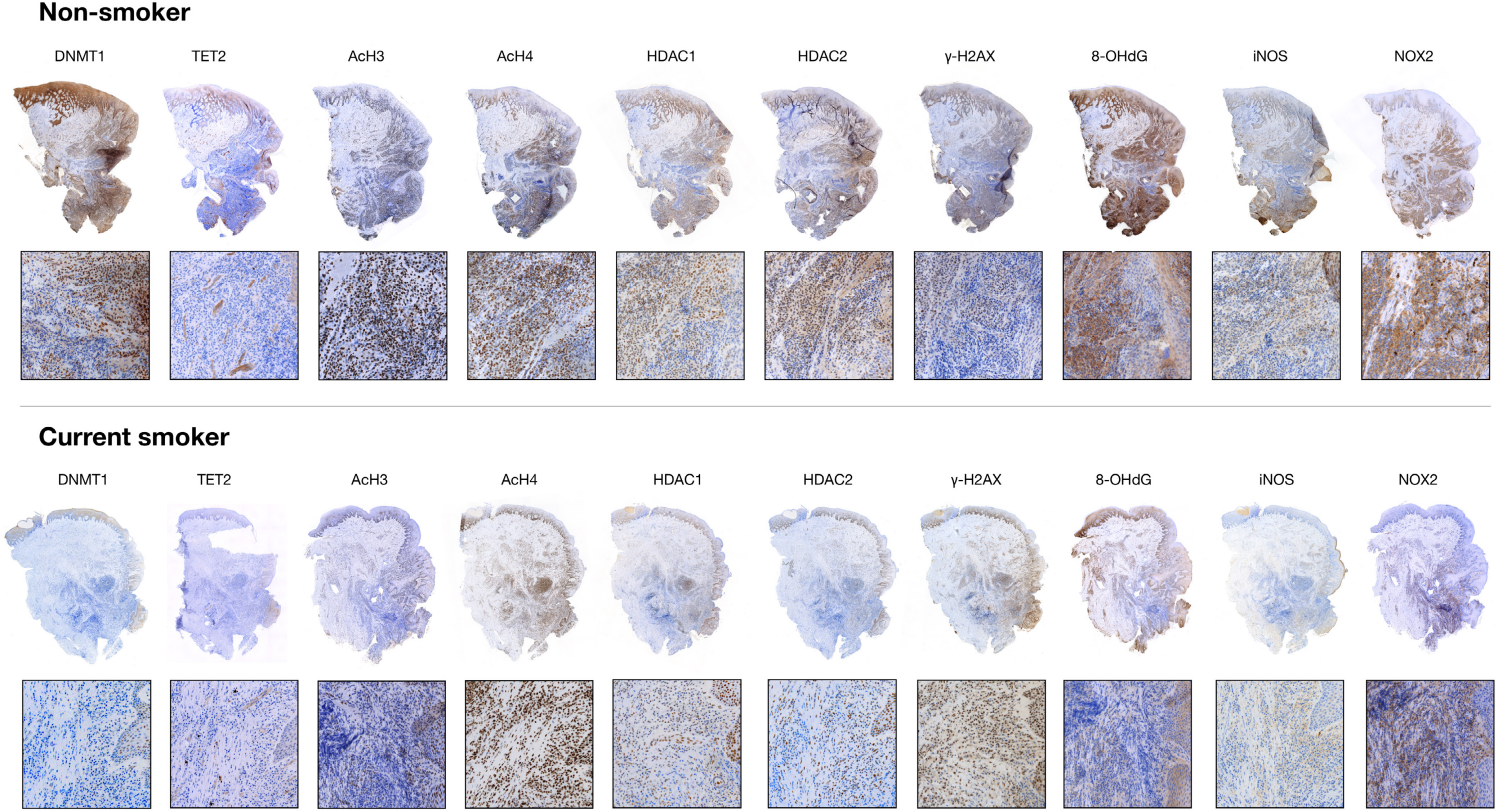
Histological sections prepared from periodontitis sites in a non‐smoker and a current smoker. Markers identified in the text. Magnification ×20 and ×400. DNA‐methyltransferase 1 (DNMT1), Ten‐eleven‐translocation 2 (TET2), Acetyl‐histone H3 (AcH3), Acetyl‐histone H4 (AcH4), Histone deacetylase 1 (HDAC1), Histone deacetylase 2 (HDAC2), Phosphorylated H2A family member X (γ‐H2AX); 8‐hydroxyguanosine (8‐OHdG), Inducible nitric oxide synthase (iNOS), NADPH‐oxidase 2 (NOX2)

Micrographs of periodontitis sites obtained from a current smoker and a non‐smoker are presented in Figure 1 and a panel of micrographs representing each cellular marker in both groups is depicted in Figure 2. In both types of specimens, a well‐defined area of ICT was identified lateral to the pocket epithelium. A gradient decline of cellular densities from the inner zone of the ICT, lateral to the pocket epithelium, to the peripheral portions of the ICT was noted in both groups. While cells positive for the AcH3, iNOS, and the NOX2 markers were evenly distributed over the ICT in both groups, cells positive for the TET2 marker were detected in the proximity to vascular units of the connective tissue compartment lateral of the pocket epithelium. DNMT1‐positive cells within the ICT were scarce in current smokers.

**FIGURE 3:**
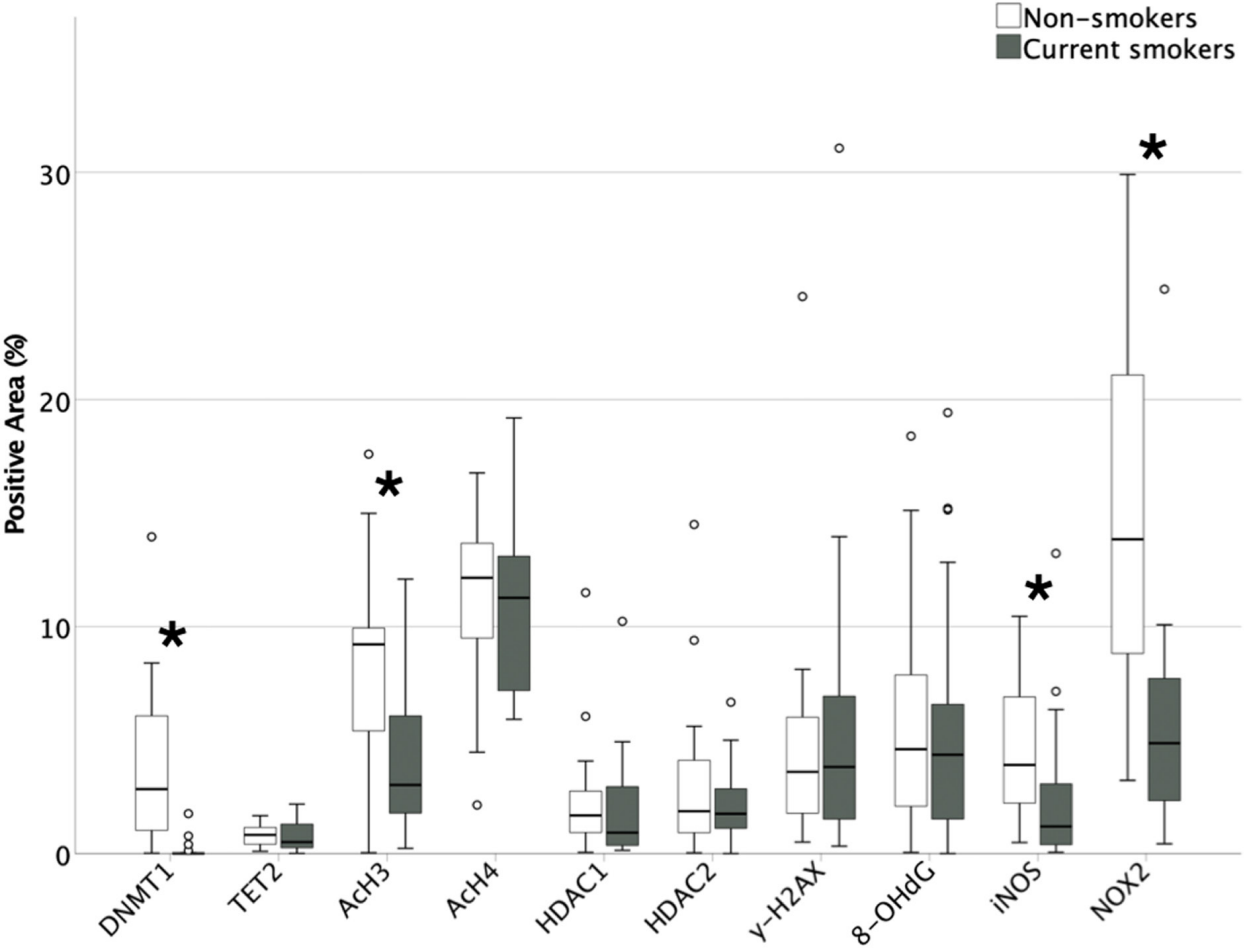
Boxplot (median value: black line, interquartile ranges: whiskers) representing the area proportions of the infiltrated connective tissue (ICT) occupied by positive cells (%) in non‐smokers (*N* = 21) and current smokers (*N* = 25). *p < .05 (Mann–Whitney *U*‐test). ○: Outlier. DNA‐methyltransferase 1 (DNMT1), Ten‐eleven‐translocation 2 (TET2), Acetyl‐histone H3 (AcH3), Acetyl‐histone H4 (AcH4), Histone deacetylase 1 (HDAC1), Histone deacetylase 2 (HDAC2), Phosphorylated H2A family member X (γ‐H2AX); 8‐hydroxyguanosine (8‐OHdG), Inducible nitric oxide synthase (iNOS), NADPH‐oxidase 2 (NOX2)

The mean total area of the ICT was 1.47 ± 0.26 mm^2^ in samples from current smokers and 1.59 ± 0.27 mm^2^ in non‐smokers. The results from the histological analysis of the area proportions of positive cells are presented in Table 2 and in Figure 3. Current smokers showed significantly lower area proportions occupied by AcH3‐, DNMT1‐, NOX2‐, and iNOS‐positive cells in the ICT than non‐smokers. The largest difference in area proportions between the two groups was noted for the DNMT1 and NOX2 markers.

**TABLE 2:** ICT total area (mm^2^) and area proportions of positive cells (%) in non‐smokers (*N* = 21) and current smokers (*N* = 25)

	ICT Tot Area (mm^2^)	Area Proportions (%)									
		DNMT1	TET2	ACH3	ACH4	HDAC1	HDAC2	γ‐H2AX	8‐OHdG	iNOS	NOX2
Non‐smokers	1.59 (0.27)	3.91 (3.68)	0.81 (0.45)	8.24 (4.43)	11.03 (4.18)	2.50 (2.85)	3.14 (3.51)	4.46 (5.14)	5.82 (5.01)	4.47 (3.01)	15.10 (7.79)
		*		*						*	*
Current smokers	1.47 (0.26)	0.13 (0.39)	0.79 (0.68)	4.34 (3.33)	10.93 (3.82)	2.04 (2.38)	2.12 (1.66)	5.59 (6.63)	5.59 (5.39)	2.36 (3.03)	5.47 (5.07)

Mean values (SD).

*p < .05 (Mann–Whitney *U*‐test).

Abbreviations: DNA‐methyltransferase 1 (DNMT1); Ten‐eleven‐translocation 2 (TET2); Acetyl‐histone H3 (AcH3); Acetyl‐histone H4 (AcH4); Histone deacetylase 1 (HDAC1); Histone deacetylase 2 (HDAC2); Phosphorylated H2A family member X (γ‐H2AX); 8‐hydroxyguanosine (8‐OHdG); Inducible nitric oxide synthase (iNOS); NADPH‐oxidase 2 (NOX2).

The results from the assessment of the total number and the relative density (number of cells/mm^2^) of positive cells within the ICT for each marker are listed in Table 3. The total number of DNMT1‐ and NOX2‐positive cells were significantly lower in specimens from smokers than from non‐smokers. The cellular densities of AcH3‐, DNMT1‐, NOX2‐, and iNOS‐positive cells were significantly lower in samples from smokers than from non‐smokers.

**TABLE 3:** Cell size (μm^2^), estimated total number of positive cells and density of positive cells (cells/mm^2^) in the ICT of non‐smokers (*N* = 21) and current smokers (*N* = 25)

Cell size	DNMT1	TET2	AcH3	AcH4	HDAC1	HDAC2	γ‐H2AX	8‐OHdG	iNOS	NOX2
	25.79	108.27	33.29	28.92	26.26	34.51	22.45	55.10	32.39	50.49
Total no. of cells										
Non‐smokers	2827 (3408)	126 (117)	5397 (5388)	8120 (6333)	1843 (3218)	1613 (2309)	1756 (2165)	1165 (1127)	1237 (1474)	3679 (2862)
	*									*
Current smokers	72 (227)	109 (121)	2738 (3565)	6409 (5453)	1398 (2179)	971 (1032)	2258 (2445)	1312 (1674)	673 (962)	1562 (1712)
Number of cells/mm^2^										
Non‐smokers	1515 (1422)	75 (42)	2475 (1330)	3815 (1445)	950 (1086)	911 (1017)	1988 (2289)	1057 (909)	1380 (929)	2990 (1542)
	*		*						*	*
Current smokers	52 (151)	73 (63)	1303 (999)	3781 (1321)	778 (908)	615 (480)	2488 (2955)	1016 (979)	729 (936)	1083 (1004)

Mean values (SD).

*p < 0.05 (Mann–Whitney *U*‐test).

Abbreviations: DNA‐methyltransferase 1 (DNMT1); Ten‐eleven‐translocation 2 (TET2); Acetyl‐histone H3 (AcH3); Acetyl‐histone H4 (AcH4); Histone deacetylase 1 (HDAC1); Histone deacetylase 2 (HDAC2); Phosphorylated H2A family member X (γ‐H2AX); 8‐hydroxyguanosine (8‐OHdG); Inducible nitric oxide synthase (iNOS); NADPH‐oxidase 2 (NOX2).

## 4 DISCUSSION

The present study evaluated differences in the cellular expression of epigenetic markers and reactive oxygen/nitrogen species in periodontitis lesions between current smokers and non‐smokers. Although the ICT of smokers and non‐smokers did not differ in size or in the expression of markers for DNA damage or oxidative stress, current smokers presented with significantly lower area proportions and densities of cells positive for the epigenetic markers DNMT1 and AcH3. In addition, periodontitis lesions in current smokers presented with a diminished antimicrobial activity, as indicated by significantly lower densities and area proportions of NOX2‐ and iNOS‐positive cells than periodontitis lesions in non‐smokers.

One of the main findings of the present study was the large difference in DNA methylation levels in periodontitis lesions between current smokers and non‐smokers, as revealed by the significantly lower density of DNMT1‐positive cells in current smokers than in non‐smokers. This observation is in accordance with data reported by Richter et al.^24^ who analyzed samples of oral masticatory mucosa from smokers and non‐smokers. Similar findings were also presented by Cho et al.^15^, ^25^ in studies on DNA methylation status in gingival biopsies from smokers and non‐smokers. On the other hand, analysis of DNA de‐methylation mechanisms, which are initiated by TET‐proteins, failed to demonstrate differences between the groups of tissue samples in the present study, as indicated by the assessment of densities of TET2‐positive cells. Thus, smokers appear to present with downregulated DNA methylation levels in inflamed gingival tissues as opposed to non‐smokers, while TET2 levels in periodontitis lesions seem to be unaffected by smoking status.

A further epigenetic mechanism investigated in the present study on potential differences between current smokers and non‐smokers was histone modification, as indicated by histone acetylation or deacetylation. Indeed, a reduction of histone acetylation in periodontitis lesions, as shown by lower densities of AcH3‐positive cells, was detected in the current smoker group. Evaluations of other markers on histone acetylation (AcH4) or histone deacetylation (HDAC1/HDAC2), however, did not disclose any differences between the two groups. Histone acetylation and histone deacetylation are associated with several cellular processes, including gene transcription, and the relevance of analyzing markers of such functions in inflamed gingival tissues was demonstrated by Cantley et al.^26^ They analyzed gingival biopsies obtained from sites with periodontitis or gingivitis and reported that HDAC levels overall were higher in periodontitis lesions and that the expression of HDAC1 in endothelial cells was the most conspicuous difference between periodontitis and gingivitis specimens. The observation of upregulated histone deacetylation in endothelial cells of periodontitis lesions by Cantley et al.^26^ is of importance in the evaluation of results from the present study. Although HDAC1‐positive cells occurred in similar densities in periodontitis lesions of current smokers and non‐smokers, data presented in the preceding report by Schmidt et al.^23^ demonstrated that the density of vascular units was smaller in periodontitis lesions of current smokers than in lesions of non‐smokers. Thus, the effect of the downregulation of histone acetylation and the unaltered expression of histone deacetylation on vascular units in periodontitis lesions of current smokers remains to be elucidated.

The present study failed to detect differences in proportions and densities of cellular markers for DNA damage (γ‐H2AX) and oxidative stress (8‐OHdG) between the two groups. Similar results were shown by Hendek et al.^27^ who compared salivary 8‐OHdG levels in smoking and non‐smoking patients with periodontitis. While both groups of patients demonstrated a significant reduction in salivary levels after non‐surgical periodontal therapy, no differences were found between the groups. Thus, in accordance with previously reported data,^28^, ^29^, ^30^ the elevated numbers and cellular densities of γ‐H2AX‐ and 8‐OHdG‐positive cells observed in the present study indicate that oxidative stress is a typical characteristic of periodontal inflammation, irrespective of the smoking status of the patient.

In addition to analysis of epigenetic and oxidative stress mechanisms, the present study also investigated cellular characteristics of antimicrobial activity in periodontitis lesions of the two groups of patients. Thus, periodontitis lesions in current smokers presented with lower densities of iNOS‐ and NOX2‐positive cells as opposed to periodontitis lesions in non‐smokers. As iNOS and NOX2 markers represent important functions of RONS of the innate host response, the findings in the present study indicate that periodontitis lesions of smokers exhibit a suppressed antimicrobial capacity. This observation is, however, in contrast with data presented by Özdemir et al.^31^ who reported that specimens representing gingivitis and healthy gingival tissue in smokers presented with higher levels of iNOS than those found in corresponding tissue samples from never‐smokers. It should be realized, however, that the study by Özdemir et al.^31^ did not include periodontitis lesions and did not assess cellular densities.

The finding in the present study on lower densities of NOX2‐positive cells in periodontitis lesions of current smokers indicates a disturbance in neutrophil functions, including the formation of neutrophil extracellular traps (NETs) and the elimination of pathogens through the “respiratory burst” process.^32^, ^33^ As NOX2 is part of the NADPH‐oxidase family, studies showing associations between genetic polymorphisms of NADPH‐oxidase and the condition “aggressive periodontitis” of the former classification are of interest (e.g., Nibali et al.^34^). Papapanou et al.^35^ analyzed soft tissue biopsies obtained from patients affected by chronic or aggressive periodontitis. It was reported that a higher expression of NAD^+^, a precursor of NADPH, was noted in aggressive periodontitis sites than in chronic periodontitis sites.

Taken together, in addition to previous findings of fewer but wider vessels in periodontitis lesions of current smokers than in non‐smokers,^23^ the results of the present study showed that important components of the host response and epigenetic mechanisms in periodontitis lesions in smokers are downregulated as opposed to lesions of non‐smokers. It is important to note, however, that differential gene expression analysis was not conducted in the present study. Furthermore, limitations concerning the interpretation of results obtained from the analysis of cellular markers in histological specimens should always be considered. In fact, the cellular expression of various markers in histological preparations does not necessarily reveal an ongoing process or molecular production. Nevertheless, since tissue‐specific variability plays an important role in epigenetic mechanisms, a strength of the present study is the analysis of the entire ICT in well‐preserved specimens. Lastly, although the power calculation was made for the preceding study on phenotype markers, the present sample size was sufficient to detect differences in selected markers between current smokers and non‐smokers.

In conclusion, while a clear linkage between epigenetics and inflammation remains to be established, the results of the present study point to an association between epigenetic modifications and severe periodontitis depending on whether or not the patients smoke tobacco products. These findings should be considered in risk assessments and classification of periodontitis patients. Clinicians need to be aware that smokers require specific attention in treatment planning and maintenance.

## Supporting information


AppendixS1‐S2

## Data Availability

The data that support the findings of this study are available from the corresponding author upon reasonable request.
