# Supplementary material for: Cellular expression of epigenetic markers and oxidative stress in periodontitis lesions of smokers and non‐smokers
Source: J Periodontal Res. 2022 Jun 29;57(5):952–9. doi: 10.1111/jre.13030 (PMC9542336; doi:10.1111/jre.13030)
Supplement: Supplementary file 1 — AppendixS1‐S2 [file JRE-57-952-s001.docx]

**Appendix**

*Table S1*. Demographic characteristics of non-smokers (N=21) and current smokers (N=25)

|  | Age | Gender  N of Female/Male  (%) | Pack-years | Cotinine  (*μ*g l^−1^) |
| --- | --- | --- | --- | --- |
| Non-smokers | 53.7 ± 12.7  [35–76] | 12/9  (57/43) | 5.6 ± 9.9 | <10 |
|  |  |  |  | * |
| Current smokers | 48.5 ± 9.2  [33–69] | 13/12  (52/48) | 25.4 ± 12.7 | - 1. ± 188.2 |

Mean values ± SD

[ ], range

**p* < .001 (Student *t*-test / Fisher’s exact test)

## *Table S2*. Characteristics of patients and biopsy sites

| Non-smokers | | | | | Current smokers | | | | |
| --- | --- | --- | --- | --- | --- | --- | --- | --- | --- |
| Patient | Age | Tooth | Site | PPD | Patient | Age | Tooth | Site | PPD |
| 1 | 40 | 27 | D | 9 | 1 | 33 | 28 | P | 6 |
| 2 | 43 | 14 | D | 9 | 2 | 41 | 17 | M | 7 |
| 3 | 45 | 23 | D | 6 | 3 | 43 | 47 | D | 7 |
| 4 | 49 | 44 | M | 9 | 4 | 45 | 46 | M | 6 |
| 5 | 53 | 26 | D | 6 | 5 | 45 | 46 | D | 7 |
| 6 | 39 | 16 | D | 6 | 6 | 46 | 25 | D | 10 |
| 7 | 46 | 26 | D | 9 | 7 | 49 | 16 | M | 9 |
| 8 | 65 | 16 | D | 8 | 8 | 51 | 14 | D | 6 |
| 9 | 62 | 26 | D | 6 | 9 | 52 | 46 | M | 6 |
| 10 | 73 | 26 | M | 9 | 10 | 53 | 27 | M | 10 |
| 11 | 58 | 34 | D | 12 | 11 | 53 | 16 | D | 7 |
| 12 | 35 | 16 | D | 9 | 12 | 57 | 17 | M | 9 |
| 13 | 38 | 13 | M | 6 | 13 | 58 | 24 | D | 8 |
| 14 | 53 | 25 | D | 6 | 14 | 59 | 26 | D | 7 |
| 15 | 71 | 13 | D | 12 | 15 | 42 | 36 | M | 8 |
| 16 | 76 | 24 | P | 7 | 16 | 69 | 14 | M | 8 |
| 17 | 43 | 27 | M | 6 | 17 | 42 | 17 | D | 7 |
| 18 | 72 | 24 | D | 7 | 18 | 44 | 36 | D | 6 |
| 19 | 54 | 17 | D | 7 | 19 | 33 | 26 | M | 8 |
| 20 | 64 | 46 | D | 6 | 20 | 50 | 16 | M | 8 |
| 21 | 48 | 26 | M | 7 | 21 | 67 | 46 | D | 9 |
|  |  |  |  |  | 22 | 49 | 22 | P | 6 |
|  |  |  |  |  | 23 | 44 | 47 | M | 8 |
|  |  |  |  |  | 24 | 53 | 27 | P | 7 |
|  |  |  |  |  | 25 | 34 | 26 | D | 6 |
|  |  |  |  |  |  |  |  |  |  |
| Mean | 53.67 |  |  | 7.71 |  | 48.48 |  |  | 7.44 |
| SD | 12.74 |  |  | 1.90 |  | 9.24 |  |  | 1.26 |

Location of the tooth site in the 1st - 4th quadrants is described using the FDI system.

Location of the biopsies at the mesial/distal/buccal or palatal aspect (M/D/B/P).

Probing pocket depth (PPD).
